# Metropolitan Hospital Sunday Fund

**Published:** 1906-08-11

**Authors:** 


### METROPOLITAN HOSPITAL SUNDAY FUND.

### COUNCIL MEETING.

A meeting of the Council of the Metropolitan Hospital Sunday Fund was held on the 2nd instant at the Mansion House, Sir Horatio Davies occupying the Chair in the absence of the Lord Mayor, the President and Treasurer.

A letter having been read from Lord Kilmorey, Chairman of the Charing Cross Hospital, expressing great regret at the recommendation to defer the award and expressing the urgent need of the hospital for ready money wherewith to carry on if wards were not to be closed; the adoption of the report was moved by Sir William Church, and seconded by Sir Savile Crossley.

Sir Edward Durning-Lawrence protested against the reduction of the grant to the Waterloo Road Women's and Children's Hospital from £250 to £46. They had worked very hard, he said, to raise the £15,000 required to pay off their debt, and he thought they had been treated very harshly.

Sir Savile Crossley said the most careful consideration had been given to the case of Charing Cross, and it was with great regret the Committee had come to the decision they had. The Women's and Children's Hospital had also been very carefully considered.

Sir William Church said he presumed Sir Edward Durning-Lawrence, as Chairman, was aware that the Women's and Children's Hospital had contravened a rule of the fund by holding a bazaar, at which they had raised £8,000 a few days before Hospital Sunday. That had been taken into consideration in making the award.

Sir Edward accepted the explanation, and mentioned that the date was not of their own fixing, but that of the Duchess of Albany, who had opened the bazaar.

Dr. Glover was concerned to see that there were 571,666 attendances in the London Hospital's out-patient department, and he thought a great many of these people might have been attended to outside the hospital.

The Chairman of the London Hospital said there was only one other hospital beside the London for the whole of ast London, and, therefore, the number who attended was ecessarily very large. They were, however, in close touch ith the Provident dispensaries, and one of the most suc-essful in all London was just opposite the hospital Whenever it was possible they referred patients to local ractitioners, and he was quite satisfied there was no abuse. He could not see what was complained of except that they were doing a lot of good.

The report was adopted.

### DISTRIBUTION COMMITTEE'S REPORT AND AWARDS.

The Committee of Distribution in submitting their Report desire to record with regret the resignation of Sir Felix Schuster Bt., who is now unable to give the necessary time to the work of the fund.

The amount of the fund to the present time is £56,303, to which has to be added the sum of £3,000, a portion of the legacy of £10,000 left to the fund by the late Mr. Herbert Lloyd, making a total of £59,303.

The Committee have, in accordance with the resolution passed at the annual meeting in 1905, had the accounts of the nursing associations employing fully trained hospital nurses before them for the first time.

This year 250 Institutions have made applications for grants from the fund, being two more than in 1905, in addition to the nursing associations.

Two hospitals which applied for grants were considered ineligible.

The Committee, though fully realising the excellent work carried on by the Cancer Hospital, have not made an award this year, the Hospital not being in want of funds at the present time.

The Committee now recommend the distribution of £57,510 to the 161 hospitals and institutions, 60 dispensaries and 26 nursing associations shown in the appendix attached hereto.

The Committee having reduced the bases of award in 37 cases, this fact was intimated to each hospital interested in accordance with Law V. Twenty deputations attended the Committee, and after hearing their cases, the final awards were determined.

The Committee recommend that the award to Charing Cross Hospital be paid when it is proved to their satisfaction that the result of the special appeal to be made next autumn will enable the committee to carry on the work of the Hospital.

The Committee had been officially informed that the Governors of the City Orthopædic Hospital on July 11 resolved to amalgamate with the Royal National Orthopædic Hospital.

The Committee recommend that the grant made last year be now paid to the City Orthopædic Hospital, without waiting for the amalgamation to be carried through.

The Committee would view with satisfaction an amalgamation between the Wimbledon and South Wimbledon Cottage Hospitals, in consideration of the facts that both Hospitals require rebuilding, that further accommodation is required in the neighbourhood, and that one central institution could be worked more economically and produce better results.

The Committee are of opinion that the Billingsgate Medical Mission should keep its medical and mission work quite distinct in its accounts, that as a dispensary it is doing good work, but that a hospital is not required in this district.

Five per cent. of the total sum collected is set apart to purchase surgical appliances (in obedience to Law XI.) in monthly proportions during the ensuing year.

In compliance with an order of the Council, and for the special use of its members, tables of statistics have been prepared as usual, showing an analysis of the number of beds in hospitals, the cost of patients both at hospitals and dispensaries, the proportionate expense of management, as well as other valuable information, and copies are sent to every member of the Council.

### PARTICULARS OF AWARDS.

Particulars of the Awards recommended by the Committee of Distribution for the year 1906.

### 31 GENERAL HOSPITALS.

Charing Cross Hospital, £1,204 13s. 9d.; French Hospital, £356 5s.; German Hospital, £590 12s. 6d.; Great Northern Central Hospital, £1,069 7s. 6d.; Guy's Hospital, £1,687 10s.; Hampstead General Hospital, £234 7s. 6d.; Italian Hospital, £206 5s.; King's College Hospital, £1,359 7s. 6d.; London Hospital, £5,437 10s.; London Homœopathic Hospital, £478 2s. 6d.; Phillips' Memorial Homœopathic Hospital, £33 15s.; London Temperance Hospital, £721 17s. 6d.; Metropolitan Hospital, £1,050; Mildmay Hospital, £281 5s.; Miller Hospital and Royal Kent Dispensary, £257 16s. 3d.; North-West London Hospital, £421 17s. 6d.; Poplar Hospital, £525; Kensington General Hospital, £159 7s. 6d.; Royal Free Hospital, £1,134 7s. 6d.; St. George's Hospital, £1,837 10s.; S.S. John and Elizabeth Hospital, £309 7s. 6d.; St. John's Hospital, Lewisham, £145 6s. 3d.; St. Mary's Hospital, £2,343 15s.; Seamen's Hospital Society, £1,378 2s. 6d.; The Middlesex Hospital and Convalescent Home, £2,100; Tottenham Hospital, £543 15s.; University College Hospital, £1,706 5s.; Walthamstow, etc., Hospital, £135 18s. 9d.; West Ham Hospital, £543 15s.; West London Hospital, £1,078 2s. 6d.; Westminster Hospital, £1,500.

### SPECIAL HOSPITALS.

#### 5 Chest Hospitals.

City of London Hospital for Diseases of the Chest, Victoria Park, £900; Hospital for Consumption, Brompton, £2,203 2s. 6d.; Mount Vernon Consumption Hospital, Hampstead, £909 7s. 6d.; Royal Hospital for Diseases of the Chest, City Road, £553 2s. 6d.; Royal National Hospital for Consumption (Ventnor), £337 10s.

#### 19 Children's Hospitals.

Alexandra Hospital for Hip Disease, W.C., £384 7s. 6d.; Banstead Surgical Home, £23 8s. 9d.; Barnet Home Hospital, £43 2s. 6d.; Belgrave Hospital for Children, S.W., £135 18s. 9d.; Cheyne Hospital for Incurable Children, S.W., £140 12s. 6d.; East London Hospital for Children, Shadwell, E., £750; Evelina Hospital for Sick Children, Southwark, S.E., £182 16s. 3d.; Home for Incurable Children, Hampstead, £79 13s. 9d.; Home for Sick Children, Sydenham, S.E., £117 3s. 9d.; Hospital for Sick Children, Great Ormond Street, W.C., £1,040 12s. 6d.; Infants' Hospital, Hampstead, Nil; Kensington, for Children, and Dispensary, £84 7s. 6d.; North-Eastern Hospital for Children, Hackney Road, N.E., £562 10s.; Paddington Green Hospital for Children, W., £187 10s.; St. Mary's Hospital, Plaistow, E., £262 10s.; St. Monica's Hospital, Brondesbury, N.W., £75; Victoria Hospital for Children, King's Road, Chelsea, S.W., £562 10s.; Victoria Home, Margate, £37 10s.; Hospital for Hip Disease, Sevenoaks, £42 3s. 9d.

#### 6 Lying-in Hospitals.

British Lying-in Hospital, Endell Street, W.C., £70 6s. 3d.; City of London Lying-in Hospital, City Road, E.C., £200; Clapham Maternity Hospital, £28 2s. 6d.; East End Mothers' Home, £75; General Lying-in Hospital, Lambeth, S.E., £28 2s. 6d.; Queen Charlotte's Lying-in Hospital, Marylebone Road, W., £468 15s.

#### 6 Hospitals for Women.

Chelsea Hospital for Women, S.W., £281 5s.; Hospital for Women, Soho Square, W., £403 2s. 6d.; Grosvenor Hospital for Women and Children, Vincent Square, S.W., £159 7s. 6d.; New Hospital for Women, Euston Road, W.C., £225; Royal Waterloo Hospital for Children and Women, Lambeth, S.E., £46 17s. 6d.; Samaritan Free Hospital, Marylebone Road, W., £421 17s. 6d.

### 26 OTHER SPECIAL HOSPITALS.

Cancer Hospital, Brompton, S.W., Nil; Middlesex Hospital, Cancer Wing, £263 8s. 9d.; London Fever Hospital,

Islington, N., £187 10s.; Gordon Hospital for Fistula, Vauxhall Bridge Road, S.W., £14 1s. 3d.; St. Mark's Hospital for Fistula, City Road, E.C., £248 8s. 9d.; National Hospital for the Diseases of the Heart, Soho Square, W., £154 13s. 9d.; Female Lock Hospital, Harrow Road, W., £253 2s. 6d.; Hospital for Epilepsy, Paralysis and other diseases of the Nervous System, Maida Vale, W., £126 11s. 3d.; National Hospital for the Paralysed and Epileptic, W.C., £1,078 2s. 6d.; West End Hospital for Diseases of the Nervous System, £290 12s. 6d.; Central London Ophthalmic Hospital, Gray's Inn Road, W.C., £159 7s. 6d.; Royal Eye Hospital, St. George's Circus, S.E., £267 3s. 9d.; Royal London Ophthalmic Hospital, City Road, E.C., £1,031 5s.; Royal Westminster Ophthalmic Hospital, Charing Cross, W.C., £159 7s. 6d.; Western Ophthalmic Hospital, Marylebone Road, W., £75; Royal National Orthopædic Hospital, Great Portland Street, W., £150; Royal Sea Bathing Hospital, Margate, £271 17s. 6d.; St. John's Hospital for Diseases of the Skin, £18 15s.; Western Skin Hospital, Great Portland Street, W., £4 13s. 9d.; St. Peter's Hospital for Stone, Covent Garden, £70 6s. 3d.; Central London Throat and Ear Hospital, Gray's Inn Road, W.C., £43 2s. 6d.; Hospital for Diseases of the Throat, Golden Square, W., £28 2s. 6d.; London Throat Hospital, Great Portland Street, W., £23 8s. 9d.; Royal Ear Hospital, Frith Street, W., £27 3s. 9d.; Royal Dental Hospital of London, W.C., £290 12s. 6d.; National Dental Hospital, 149, Great Portland Street, W., £15.

### 32 CONVALESCENT HOSPITALS.

Metropolitan Convalescent Institution, Walton and Broadstairs, £628 2s. 6d.; Metropolitan Convalescent Institution, Bexhill, £412 10s.; All Saints' Convalescent Hospital, Eastbourne, £375; All Saints' Convalescent Home, St. Leonards-on-Sea, £23 8s. 9d.; Ascot Priory Convalescent Home, £70 6s. 3d.; Brentwood Convalescent Home for Children, £9 7s. 6d.; Charing Cross Hospital Convalescent Home, Limpsfield, £70 6s. 3d.; Chelsea Hospital for Women Convalescent Home, St. Leonards, £42 3s. 9d.; Deptford Medical Mission Convalescent Home, Bexhill, £18 15s.; Fairlight Convalescent Home, Hastings, £23 8s. 9d.; Friendly Societies' Convalescent Home, Dover, £84 7s. 6d.; Mrs. Gladstone Convalescent Home, Mitcham, £89 1s. 3d.; Hahnemann Convalescent Home, Bournemouth, £28 2s. 6d.; Hanwell Convalescent Home, £15 18s. 9d.; Hendon, Ossulston Home, £53 8s. 9d.; Herbert Convalescent Home, Bournemouth, £28 2s. 6d.; Herne Bay Baldwin-Brown Convalescent Home, £56 5s.; Homœopathic Hospital Convalescent Home, Eastbourne, £23 8s. 9d.; Hemel Hempstead Convalescent Home, £43 2s. 6d.; Incorporated Morley and Bevan Convalescent Home, £281 5s.; Mrs. Kitto's Convalescent Home, Reigate, £46 17s. 6d.; London Hospital Convalescent Home, Tankerton, £67 10s.; Mary Wardell Convalescent Home for Scarlet Fever, £56 5s.; Police Seaside Home, Brighton, £46 17s. 6d.; St. Andrew's Convalescent Home, Clewer, £93 15s.; St. Andrew's Convalescent Home, Folkestone, £168 15s.; St. John's Home for Convalescent and Crippled Children, Brighton, £32 16s. 3d.; St. Joseph's Convalescent Home, Bournemouth, £56 5s.; St. Leonards-on-Sea Convalescent Home for Poor Children, £95 12s. 6d.; St. Mary's Convalescent Home, Shortlands, £20 12s. 6d.; St. Michael's Convalescent Home, Westgate-on-Sea, £32 16s. 3d.; Seaside Convalescent Hospital, Seaford, £93 15s.

### 24 COTTAGE HOSPITALS.

Acton Cottage Hospital, £56 5s.; Beckenham Cottage Hospital, £75; Billingsgate Medical Mission Hospital, £46 17s. 6d.; Blackheath and Charlton Cottage Hospital, £79 13s. 9d.; Bromley, Kent, Cottage Hospital, £117 3s. 9d.; Bushey Heath Cottage Hospital, £34 13s. 9d.; Canning Town Cottage Hospital, £56 5s.; Chislehurst, Sidcup and Cray Valley Cottage Hospital, £60 18s. 9d.; Coldash Cottage Hospital, £20 12s. 6d.; East Ham Cottage Hospital, £72 3s. 9d.; Eltham Cottage Hospital, £45; Enfield Cottage Hospital, £65 12s. 6d.; Epsom and Ewell Cottage Hospital, £50 12s. 6d.; Hounslow Cottage Hospital, £42 3s. 9d.; Dartford Cottage Hospital, £84 7s. 6d.; Kingston, Victoria Hospital, £62 16s. 3d.; Mildmay Cottage Hospital, £23 8s. 9d.; Reigate and Redhill Cottage Hospital, £103 2s. 6d.; Sidcup Cottage Hospital, £32 16s. 3d.; Tilbury (Passmore Edwards) Cottage Hospital, £46 17s. 6d.; Willesden Cottage Hospital, £103 2s. 6d.; Wimbledon Cottage Hospital, £46 17s. 6d.; Wimbledo[illegible] South Cottage Hospital, £46 17s. 6d.; Woolwich and Plum[illegible]stead Cottage Hospital, £51 11s. 3d.

### 15 INSTITUTIONS.

Bolingbroke Hospital, £271 17s. 6d.; Hospital for I[illegible]valid Gentlewomen, Harley Street, £93 15s.; S. Saviour[illegible] Hospital and Nursing Home, £94 13s. 9d.; National San[illegible]torium for Consumption, Bournemouth, £93 15s.; Invali[illegible] Asylum, Stoke Newington, £37 10s.; Firs Home, Bourn[illegible]mouth, £28 2s. 6d.; St. Catherine's Home, Ventno[illegible] £23 8s. 9d.; Friedenheim Hospital for the Dying, £300[illegible] Oxygen Home, Fitzroy Square, £37 10s.; Santa Clau[illegible] Home, Highgate, £59 1s. 3d.; Free Home for Dying, Clapham, £121 17s. 6d.; St. Luke's House, Pembridge Square[illegible] W., £112 10s.; Royal Mineral Water Hospital, Bat[illegible] £46 17s. 6d.; Eversfield, St. Leonards, Nil; St. Winifred's Home, Holloway, £56 5s.

### 60 DISPENSARIES.

Battersea Provident Dispensary, £112 10s.; Blackfriars Provident Dispensary, £18 15s.; Bloomsbury Provident Dispensary, £14 1s. 3d.; Brixton, etc., Dispensary, £49 13s. 9d.; Brompton Provident Dispensary, £18 15s.; Buxton Street Dispensary, £18 15s.; Camberwell Provident Dispensary, £77 16s. 3d.; Camden Town Provident Dispensary £15; Chelsea, Brompton and Belgrave Dispensary, £44 1s. 3d.; Chelsea Provident Dispensary, £13 2s. 6d.; Childs Hill Provident Dispensary, £15 18s. 9d.; City Dispensary, £51 11s. 3d.; City of London and East London Dispensary, £9 7s. 6d.; Clapham General and Provident Dispensary, £22 10s.; Deptford Medical Mission, £23 8s. 9d.; Eastern Dispensary, £34 13s. 9d.; East Dulwich Provident Dispensary, £38 8s. 9d.; Farringdon General Dispensary, £42 3s. 9d.; Finsbury Dispensary, £46 17s. 6d.; Forest Hill Provident Dispensary, £37 10s.; Greenwich Provident Dispensary, £31 17s. 6d.; Hackney Provident Dispensary, £16 17s. 6d.; Hampstead Provident Dispensary, £58 2s. 6d.; Holloway and North Islington Dispensary, £42 3s. 9d.; Islington Dispensary, £56 5s.; Islington Medical Mission, £38 8s. 9d.; Kensal Town Provident Dispensary, £15 18s. 9d.; Kennington and Vauxhall Provident Dispensary, £13 2s. 6d.; Kentish Town Medical Mission, £19 13s. 9d.; Kilburn, Maida Vale, and S. John's Wood Dispensary, £38 8s. 9d.; Kilburn Provident Medical Institution, £46 17s. 6d.; London Dispensary, Spitalfields, £14 1s. 3d.; London Medical Mission, Endell Street, £116 5s.; Margaret Street, for Consumption, £21 11s. 3d.; Metropolitan Dispensary, £58 2s. 6d.; Mildmay Medical Mission Dispensary, £23 8s. 9d.; Notting Hill Provident Dispensary, £13 2s. 6d.; Paddington Provident Dispensary, £31 17s. 6d.; Public Dispensary, Clare Market, W.C., £35 12s. 6d.; Queen Adelaide's Dispensary, £29 1s. 3d.; Royal General Dispensary, £23 8s. 9d.; Royal Pimlico Provident Dispensary, £41 5s.; Royal South London Dispensary, £46 17s. 6d.; St. George's and St. James's Dispensary, £46 17s. 6d.; St. George's, Hanover Square, Provident Dispensary, £28 2s. 6d.; St. John's Wood Provident Dispensary, £45 18s. 9d.; St. Marylebone General Dispensary, £47 16s. 3d.; St. Pancras and Northern Dispensary, £30; St. Pancras Medical Mission, £19 13s. 9d.; South Lambeth, Stockwell, and North Brixton Dispensary, £36 11s. 3d.; Stamford Hill, Stoke Newington Dispensary, £56 5s.; Tower Hamlets Dispensary, £43 2s. 6d.; Walworth Provident Dispensary, £15; Wandsworth Common Provident Dispensary, £14 1s. 3d.; Westbourne Provident Dispensary, £16 17s. 6d.; Western Dispensary, £62 16s. 3d.; Western General Dispensary, £104 1s. 3d.; Westminster General Dispensary, £46 17s. 6d.; Whitechapel Provident Dispensary, £32 16s. 3d.; Woolwich Provident Dispensary, £24 7s. 6d.

### 26 NURSING ASSOCIATIONS.

Aldgate Freedom, £5; Aldgate Lordship, £5; Belvedere and Abbey Wood, £5; Brixton, £20; Central St. Pancras, £15; Hackney, £20; Hammersmith and Fulham, £35; Hampstead, £15; Kensington, £40; Kilburn, £15; Kingston, £75; Metropolitan (Bloomsbury), £50; St. Olave's (Bermondsey), £30; Paddington and Marylebone, £35; Plaistow, £85; Rotherhithe, £10; Shoreditch, £55; South London (Battersea), £45; Tottenham, £5; Southwark, £30; South Wimbledon, £15; Westminster, £25; Woolwich, Plumstead, and Charlton, £30; East London, £150; North London, £50; London District, £360.

